# Comparison of extraocular and intraocular pressure transducers for measurement of transient intraocular pressure fluctuations using continuous wireless telemetry

**DOI:** 10.1038/s41598-020-77880-8

**Published:** 2020-12-01

**Authors:** Jessica V. Jasien, Ye Emma Zohner, Sonia Kuhn Asif, Lindsay A. Rhodes, Brian C. Samuels, Christopher A. Girkin, Jeffrey S. Morris, J. Crawford Downs

**Affiliations:** 1 Vision Science Graduate Program, School of Optometry, University of Alabama at Birmingham, Birmingham, USA; 2 Rice University, Houston, TX USA; 3 Department of Ophthalmology and Visual Sciences, School of Medicine, University of Alabama at Birmingham, VH 390B | 1670 University Blvd., Birmingham, AL 35294 USA; 4 Perelman School of Medicine, University of Pennsylvania, Philadelphia, PA USA

**Keywords:** Biomedical engineering, Experimental models of disease, Data acquisition, Data processing

## Abstract

The optimal approach for continuous measurement of intraocular pressure (IOP), including pressure transducer location and measurement frequency, is currently unknown. This study assessed the capability of extraocular (EO) and intraocular (IO) pressure transducers, using different IOP sampling rates and duty cycles, to characterize IOP dynamics. Transient IOP fluctuations were measured and quantified in 7 eyes of 4 male rhesus macaques (NHPs) using the Konigsberg EO system (continuous at 500 Hz), 12 eyes of 8 NHPs with the Stellar EO system and 16 eyes of 12 NHPs with the Stellar IO system (both measure at 200 Hz for 15 s of every 150 s period). IOP transducers were calibrated bi-weekly via anterior chamber manometry. Linear mixed effects models assessed the differences in the hourly transient IOP impulse, and transient IOP fluctuation frequency and magnitude between systems and transducer placements (EO versus IO). All systems measured 8000–12,000 and 5000–6500 transient IOP fluctuations per hour > 0.6 mmHg, representing 8–16% and 4–8% of the total IOP energy the eye must withstand during waking and sleeping hours, respectively. Differences between sampling frequency/duty cycle and transducer placement were statistically significant (*p* < 0.05) but the effect sizes were small and clinically insignificant. IOP dynamics can be accurately captured by sampling IOP at 200 Hz on a 10% duty cycle using either IO or EO transducers.

## Introduction

Glaucoma is an ocular neurodegenerative disease that causes irreversible blindness and affects over 70 million people worldwide^1^. Intraocular pressure (IOP) is a known risk factor for glaucoma and lowering IOP is the only proven treatment for the disease^2–4^. Hence, IOP measurement has become a mainstay for assessment of both glaucoma risk and treatment efficacy. Unfortunately, IOP is sampled infrequently in clinical practice, with snapshot measurements generally obtained only during office hours as a single measurement every few months^5–8^. IOP is very dynamic and changes from day-to-day, minute-to-minute, and second-to-second. Transient IOP fluctuations comprise a significant portion of the total IOP-related energy that the eye must absorb^8^. Current snapshot IOP measurements most commonly used in clinical settings provide an inaccurate assessment of mean IOP over time and little information on transient IOP fluctuations. The study of short- and long-duration IOP fluctuations has emerged as an area of intense interest due to their potentially critical role in ocular physiology and diseases such as glaucoma^9–14^. Knowledge of the true character of IOP in humans and how it affects ocular tissues is limited due to the lack of continuous IOP monitoring technologies for patients, so there is a critical need for IOP telemetry.

The eye is a small, pressurized, soft tissue organ encased in a bony orbit. The exposed portion of the globe is dominated by the clear cornea and the optical pathway, which makes it difficult to design IOP telemetry devices that do not obstruct vision. There are several clinical devices available for telemetric IOP measurement or assessment, each of which use different approaches and have noted strengths and weaknesses. Existing telemetry systems have a wide range of sampling frequencies and duty cycles that may or may not capture the transient IOP fluctuations occurring in the eye^15,16^. None of the current devices capture true transient IOP fluctuations and hence cannot fully characterize IOP dynamics, although it remains unknown if transient IOP fluctuations play a role in glaucoma pathophysiology. Little is known about the design constraints associated with telemetric measurement of the pressure inside a rapidly moving organ like the eye. Given the limitations of current IOP telemetry approaches and the lack of accepted design and IOP sampling criteria, there is some debate as to how to best optimize IOP sensor design for telemetry. One approach is to place a sensor inside the eye, which is the gold standard for pressure measurement. However, miniaturization to the extent necessary to accommodate a minimally invasive surgical approach sacrifices measurement accuracy and results in greater long-term error through transducer drift. Powering an intraorbital device that measures transient IOP fluctuations is also a difficult engineering problem, as high sampling frequencies and continuous operation require too much power and generate too much heat to be practical. Thus, minimization of the IOP sampling frequency and the on/off duty cycle, while faithfully capturing IOP dynamics, are necessary for successful implementation. Placing the IOP transducer in an extraocular reservoir connected to the eye via a tube has measurement accuracy advantages, but a tube-based approach has the potential to damp and/or alter transient IOP fluctuation assessment through reservoir system elasticity and tube bending artefact.

We have developed and validated three wireless implantable IOP telemetry systems for research use, two that measure IOP using a tube inserted into the anterior chamber connected to an extraocular (EO) pressure transducer mounted in the orbital wall (Konigsberg Instruments EO or TSE-Systems Stellar EO), and a third where an intraocular (IO) pressure transducer was implanted in the anterior chamber of the eye (TSE-Systems Stellar IO). The purpose of the study was to compare the measurements of transient IOP fluctuations obtained using either an IO pressure transducer placed directly in the eye, or with an EO transducer mounted remotely and connected to the eye via a fluid-filled tube. Data transmission requires significant energy and generates heat, so IOP telemetry systems must grapple with the trade-offs associated with providing sufficient IOP sampling to capture IOP dynamics while minimizing data transmission. Hence, the data were analyzed to determine if continuous data sampling at 500 Hz (Konigsberg EO) yielded significantly greater information about IOP dynamics and transient IOP fluctuations compared to a 10% duty cycle in which IOP was measured at 200 Hz for 15 s out of every 150 s (TSE-Stellar EO and IO).

## Results

### Analysis of all three telemetry systems

**Figure 1:**
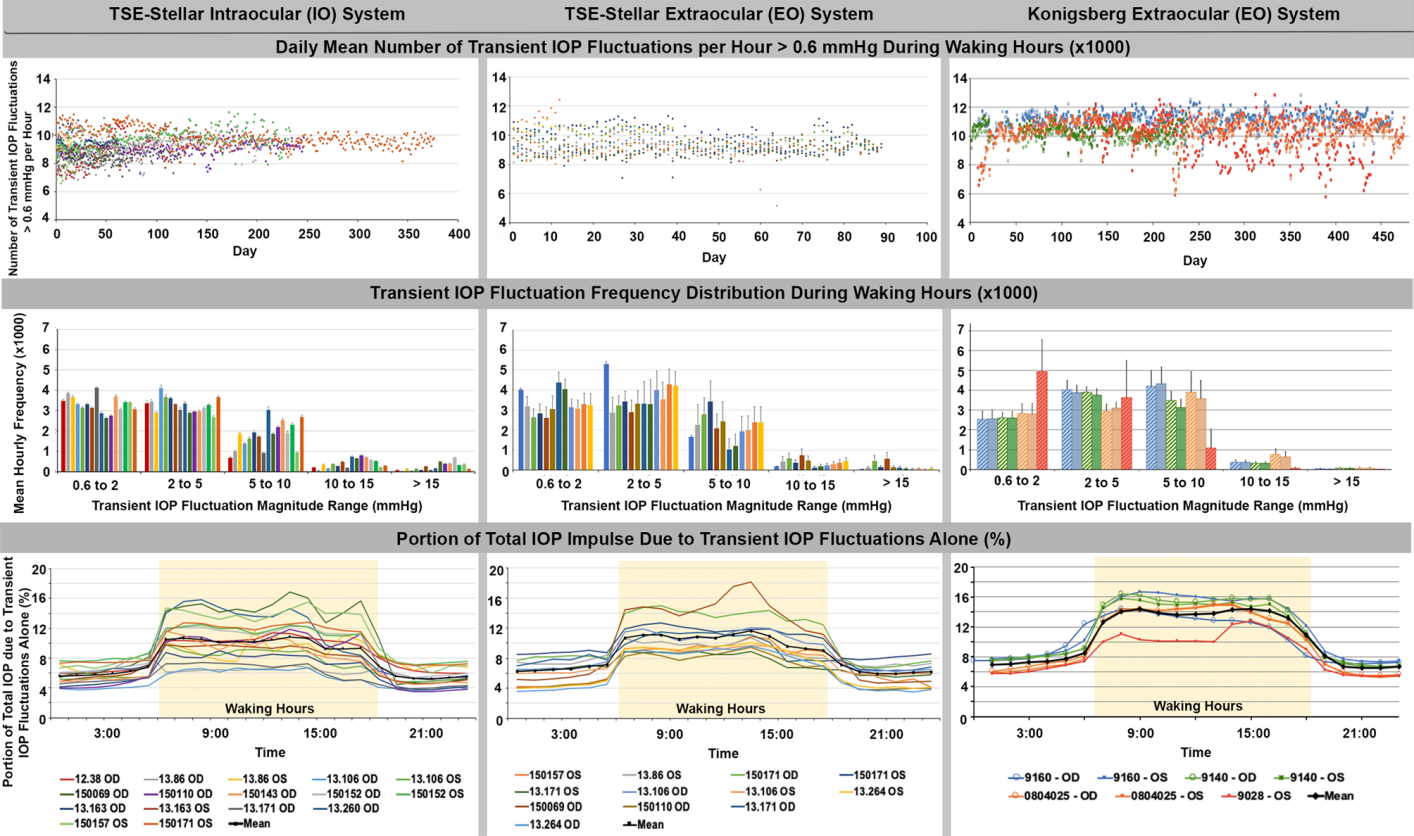
Data comparison between the TSE-Stellar IO **(left)** and TSE-Stellar EO **(middle)** and Konigsberg EO **(right**^8^, adapted from Turner et al. with permission**)** transducers. **(Top)** The daily mean number of transient IOP fluctuations per hour over 0.6 mmHg in magnitude during waking hours (6AM-6PM) plotted over the entire data acquisition period in each eye. Each data point represents one day of data, presented as the mean number of transient IOP fluctuations per hour during the 12 waking hours. The eye must withstand ~ 10,000 transient IOP fluctuations every hour during waking hours. **(Middle)** The mean hourly frequency distribution of transient IOP fluctuation magnitude during waking hours for each eye binned by the magnitude for all data collected (error bars are standard deviation). **(Bottom)** Mean hourly IOP transient impulse, plotted as percentage of total IOP impulse, for all eyes of all animals. The plotted values are a measure of the amount of energy the eye must withstand from transient IOP fluctuations relative to the total IOP energy the eye must absorb. Waking hours are highlighted.

**Figure 2:**
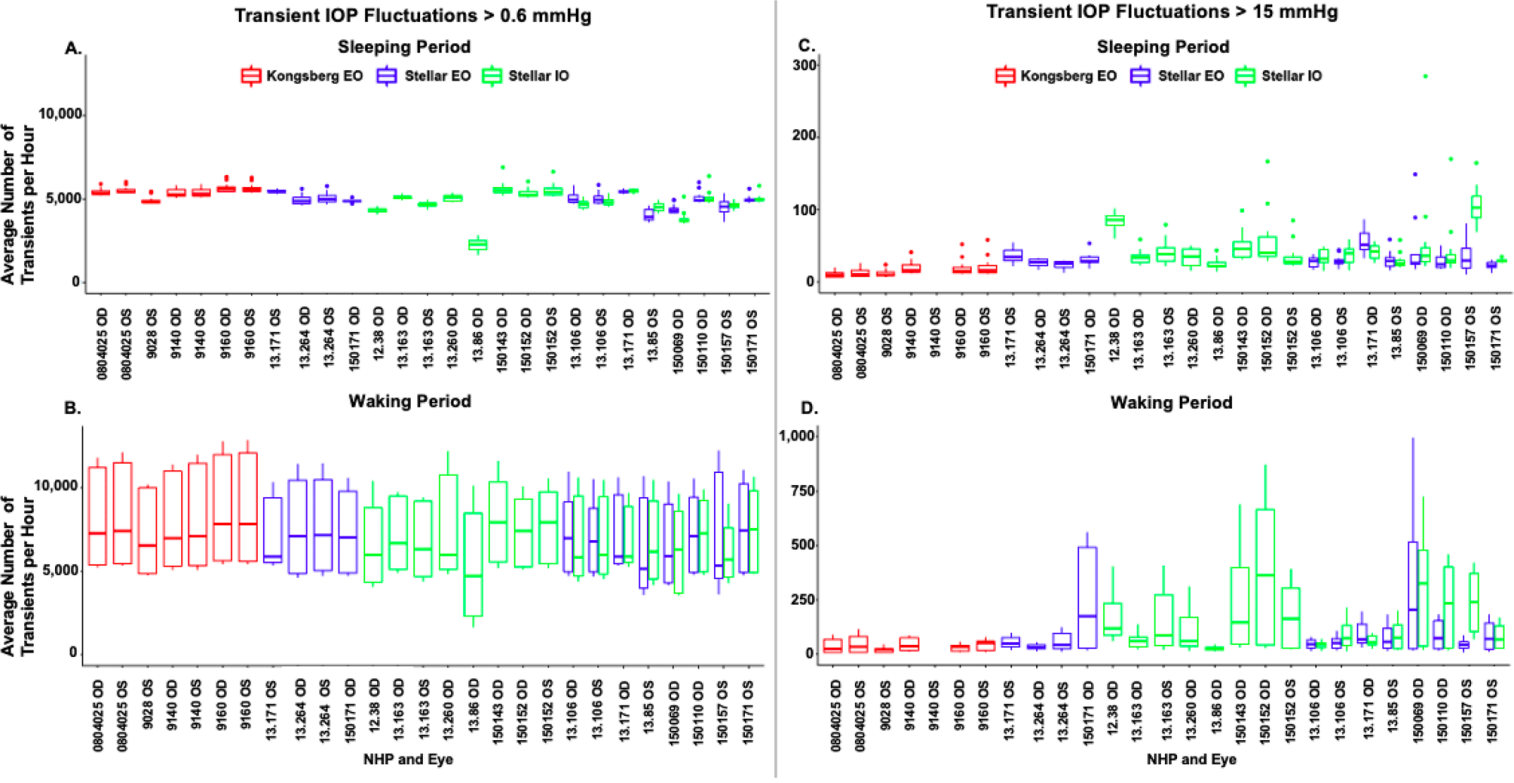
Boxplots comparing the frequency distributions of the mean number of transient IOP fluctuations per hour in each of the implant types (red, Konigsberg EO; blue, Stellar EO; green, Stellar IO) by NHP and eye in two magnitude bins (> 0.6 mmHg and > 15 mmHg above baseline IOP). **(A)** Average number of transient IOP fluctuations > 0.6 mmHg during the sleeping period for all animals **(B)** Average number of transient IOP fluctuations > 0.6 mmHg during the waking period for all animals **(C)** Average number of transient IOP fluctuations > 15 mmHg during the sleeping period for animals **(D)** Average number of transient IOP fluctuations > 15 mmHg during the waking period for all animals. The central thick line in the box represents the median number of peaks, the box extents represent the central 50% of the data, the bars represent the minimum and maximum, and the dots represent the outliers.

The intraocular Stellar IO telemetry system and the tube-based Konigsberg EO and Stellar EO telemetry systems measured 8000–12,000 transient IOP fluctuations per hour > 0.6 mmHg above momentary baseline during waking hours and 5000–6500 fluctuations/hour during sleeping hours (Figs. 1,2; Table 1). These transient IOP fluctuations represented 8–16% of the total IOP energy the eye must withstand during waking hours, and 4–8% during sleeping hours (Figs. 1, 2; Table 1).

**Table 1:** **(top)** Model-based mean number of transient IOP fluctuations per hour for the sleep and wake periods by system with 95% confidence intervals **(bottom)** and model-based mean relative contribution of transient IOP fluctuations to total IOP for the sleep and wake periods by system with 95% confidence intervals.

Magnitude–period	Stellar IO	Stellar EO	Konigsberg EO
**Mean frequency of transient IOP fluctuations per hour for sleep/wake periods by system**			
> 0.6 mmHg—Sleep	4635 ± 2046	4726 ± 2020	5548 ± 1029
> 0.6 mmHg—Wake	9372 ± 2882	9502 ± 2835	10,654 ± 1396
> 15 mmHg—Sleep	44 ± 162	39 ± 149	4 ± 23
> 15 mmHg—Wake	165 ± 310	154 ± 294	68 ± 92
**Mean contribution of transient IOP fluctuations to total IOP for Sleep/Wake By System (%)**			
Sleep	5.6 ± 4.8	5.8 ± 4.8	6.4 ± 2.4
Wake	10.3 ± 6.3	10.6 ± 6.2	11.3 ± 3

Statistical analyses showed that the difference in the frequency of transient IOP fluctuations detected by the three implant systems within NHP is significant (*p* < 0.05) for the > 0.6 mmHg magnitude bins, but the effect size was very small, on the order of 20 times smaller than the differences in these parameters between the wake and sleep periods. The Konigsberg EO system detected a significantly smaller number of transient IOP fluctuations > 15 mmHg in magnitude compared to the Stellar EO and IO systems. Similarly, the difference in the mean relative contribution of transient IOP fluctuations to total IOP by system was significant (*p* < 0.05) but with effect sizes on the order of ~ 10 times smaller than the differences seen between the wake and sleep periods.

### Analysis of eyes implanted with both the Stellar IO and Stellar EO telemetry systems

**Figure 3:**
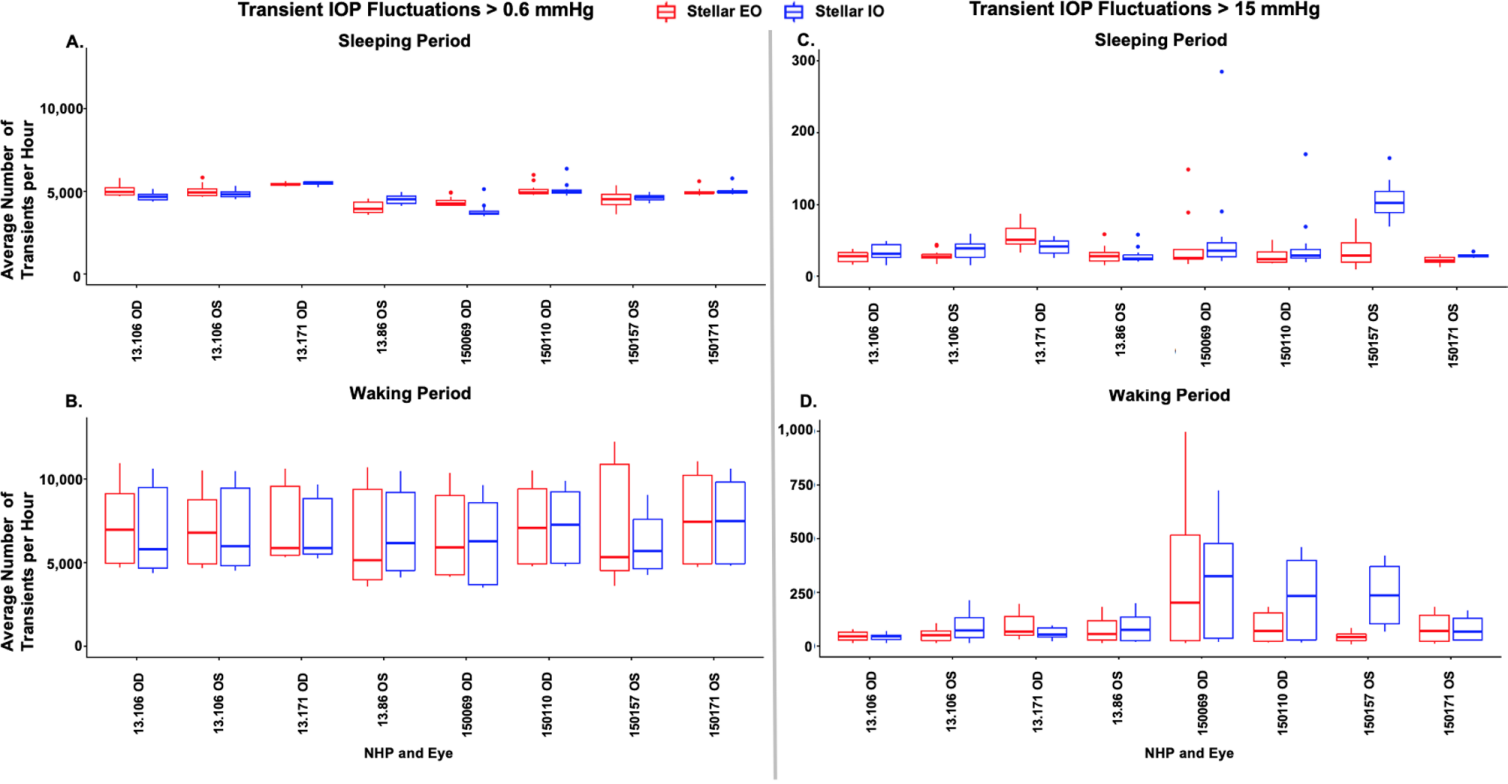
Boxplots comparing the distributions of the mean number of transient IOP fluctuations per hour in 8 eyes of 7 NHPs implanted with each the Stellar EO and Stellar IO systems (red, Stellar EO; blue, Stellar IO) by NHP and eye in two magnitude bins (> 0.6 mmHg and > 15 mmHg above baseline IOP). **(A)** Average number of transient IOP fluctuations > 0.6 mmHg during the sleeping period for all animals **(B)** Average number of transient IOP fluctuations > 0.6 mmHg during the waking period for all animals **(C)** Average number of transient IOP fluctuations > 15 mmHg during the sleeping period for animals **(D)** Average number of transient IOP fluctuations > 15 mmHg during the waking period for all animals. The central thick line in the box represents the median number of peaks, the box extents represent the central 50% of the data, the bars represent the minimum and maximum, and the dots represent the outliers.

Eight eyes of seven NHPs were implanted both the TSE-Stellar IO and EO systems; in these animals the IO system was removed, then the EO system was implanted in a replacement procedure. This allowed direct comparisons of the Stellar IO and EO systems in the same eyes. Statistical analyses showed that the difference in the frequency of transient IOP fluctuations detected by the Stellar IO and EO implant systems within eyes is significant (*p* < 0.05) for both the > 0.6 and > 15 mmHg magnitude bins, but again the effect size was very small, on the order of 15 times smaller than the differences in these parameters between the wake and sleep periods. Similarly, the difference in the mean relative contribution of transient IOP fluctuations to total IOP by system was significant (*p* < 0.05) but with effect sizes on the order of ~ 10 times smaller than the differences seen between the wake and sleep periods (Figs. 1, 3; Table 2).

**Table 2:** Data for the subset of 8 eyes of 7 NHPs in which both the Stellar IO and EO systems were implanted in series.

Magnitude and period	Stellar IO	Stellar EO
**Mean frequency of transient IOP fluctuations per hour for sleep/wake periods by system**		
> 0.6 mmHg Sleep	4587 ± 581	4755 ± 534
> 0.6 mmHg Wake	8858 ± 730	9091 ± 659
> 15 mmHg Sleep	55 ± 75	39 ± 60
> 15 mmHg Wake	197 ± 134	167 ± 115
**Mean contribution of transient IOP fluctuations to total IOP for sleep/wake by system (%)**		
Sleep	6.5 ± 2.1	6.3 ± 2
Wake	10.8 ± 2.5	10.5 ± 2.3

**(top)** Mean number of transient IOP fluctuations per hour for the sleep and wake periods by system with 95% confidence intervals and **(bottom)** the mean relative contribution of transient IOP fluctuations to total IOP for the sleep and wake periods by system with 95% confidence intervals.

## Discussion

Analysis of IOP dynamics in the 8 eyes that were implanted with both the TSE-Stellar IO and EO systems showed that both approaches capture transient IOP fluctuations similarly, with no apparent disadvantages of measuring IOP using a remote transducer connected to the eye via a fluid-filled tube. The EO approach measured significantly greater transient IOP fluctuations overall but fewer IOP fluctuations > 15 mmHg (Table 2), but the magnitude of these differences are unlikely to be clinically meaningful, especially in light of the data showing that the contribution of transient IOP fluctuations to total IOP is very similar across systems. It is unclear why the Konigsberg EO system measured significantly fewer transient IOP fluctuations > 15 mmHg in magnitude per hour. This could be due to differences in the system itself, reduced responsiveness due to the larger mass of the sensing membrane, or the data acquisition system, all of which were different from both Stellar systems. The differences between the sleeping and waking periods provide a good barometer for the differences one can easily observe in the outcome variables; all the significant differences in the outcome variables due to system or transducer location were very small compared to the sleep/wake difference. We also found that transient IOP fluctuations can be accurately assessed by measuring IOP at 200 Hz on a 10% duty cycle, showing that continuous measurement of IOP at high sampling frequencies is not necessary to characterize IOP dynamics.

In prior clinical studies, the role of “IOP fluctuations” in glaucoma pathogenesis and progression have largely been investigated using snapshot IOP measurements, most often taken from one hour to months apart^17–20^. There have been few studies directly linking transient IOP fluctuations to glaucoma. In a recent clinical study using the Triggerfish contact lens sensor (CLS) however, greater magnitudes of transient circum-limbal corneal stretch (presumably associated with transient IOP fluctuations) was associated with greater visual field deterioration in glaucoma patients^21^. These results should be viewed with caution, as it is unclear if this result was due to larger magnitude transient IOP fluctuations, or larger corneal stretch fluctuations in a more compliant ocular coat (a biomechanical factor). The need for accurate characterization of IOP dynamics and transient IOP fluctuations is mounting due to recent studies suggesting that transient IOP fluctuations may play an important role in both ocular homeostasis and the pathophysiologic changes associated with glaucoma^9–14^. Current FDA- and CE-approved devices are not sufficient to accurately assess IOP dynamics. The Sensimed Triggerfish CLS (Sensimed SA, Lausanne, Switzerland) measures circum-limbal corneal stretch presumably induced by changes in IOP in millivolt equivalent units. Thus, the Triggerfish CLS captures fluctuations of the biomechanical stretch of the ocular coat but does not measure IOP directly since no calibration method has been devised^16^. The Implandata EyeMate (Impandata Ophthalmic Products GmbH, Hanover, Germany) is a micro-electromechanical system (MEMS)-based, silicone-encapsulated ring that is implanted in the ciliary sulcus after lens replacement^22^; despite its ability to measure IOP directly and be calibrated against tonometry, the EyeMate has not been proven to be accurate over long periods of time^23^. Moreover, the EyeMate implant transmits mean IOP data only, does not measure IOP continuously, and cannot capture transient IOP fluctuations. Further, the EyeMate device is placed during cataract surgery, and many patients do not need this procedure for glaucoma treatment, although it is common in elderly patients who are also most at risk for glaucoma. Hence, there is a critical need to understand the design parameters for telemetry systems capable of fully quantifying IOP dynamics.

The results of this study should be viewed in light of the following limitations. First, there is a wide range of days of data available from each system, so the study was statistically imbalanced. However, the quantity of data available is orders of magnitude greater than any previous study, and the statistical analyses took the data imbalance into account. Similarly, there was also an imbalance in the number of animals implanted with each system due to the retrospective nature of the analysis. However, the data are consistent between animals across systems and approaches, as shown by the tables, figures and in the statistical analyses. Eight eyes of 7 NHPs had two implantation surgeries, the first to place the Stellar IO transducer and a second to remove the IO transducer and place the Stellar EO transducer. Scarring associated with the second procedure may have changed the stiffness of the sclera at the surgical site, thereby altering the magnitude of transient IOP fluctuations in the eye. However, the results show that transient IOP fluctuations captured in these eyes were comparable between systems, indicating that this effect was small if present. Finally, the study was conducted in NHPs rather than human patients, and so our results may not translate directly to human IOP telemetry systems. That said, NHPs have eyes that are remarkably similar to humans and are the most fidelic of the animal models of ocular health and disease. Hence, while the exact parameter values may not match those captured in humans in the future when IOP telemetry becomes available, the general conclusions of this study should translate well.

In conclusion, transient IOP fluctuations can be accurately captured using either a pressure transducer placed directly in the anterior chamber of the eye, or with a transducer mounted remotely and connected to the eye via a fluid-filled tube. In addition, IOP measurements captured at 200 Hz on a 10% duty cycle are sufficient to accurately quantify IOP dynamics.

## Methods

### Animals

All animals were treated in accordance with the ARVO Statement for the Use of Animals in Ophthalmic and Vision Research under a protocol approved and monitored by the UAB Institutional Animal Care and Use Committee. Seventeen adult male rhesus macaques (4–6 years old) (Table 3) with no ocular abnormalities implanted with a wireless telemetry system were used for extended data collection. Transient IOP fluctuations were measured and quantified in awake and behaving animals. Data were collected in 7 eyes of 4 male rhesus macaques (NHPs) using the Konigsberg EO system and 16 eyes of 12 male NHPs with pressure transducers inserted directly in the anterior chamber of the eye (Stellar IO). In 8 eyes of 7 NHPs, transient IOP fluctuations were also quantified using the Stellar EO system after removal of the first Stellar IO system and placement of the Stellar EO system in a second surgical implantation (Table 3). All animals were kept on a 6 AM–6 PM light–dark cycle and fed at approximately 6 AM and 2 PM daily. All animals received water ad libitum through a continuous feed, where food and water intake were not measured for this study.

**Table 3:** Animal demographics; separated by telemetry system and extraocular (EO) or intraocular (IO) transducer location and length of time (days) data were collected by system and transducer.

NHP	Eye	System	IO or EO	Days
9160	OD	Konigsberg	EO	412
9160	OS	Konigsberg	EO	412
9140	OD	Konigsberg	EO	199
9140	OS	Konigsberg	EO	181
804,025	OD	Konigsberg	EO	451
804,025	OS	Konigsberg	EO	451
9028	OS	Konigsberg	EO	300
13.163	OD	Stellar	IO	68
13.163	OS	Stellar	IO	68
150,143	OD	Stellar	IO	22
150,152	OD	Stellar	IO	207
150,152	OS	Stellar	IO	233
12.38	OD	Stellar	IO	150
13.26	OD	Stellar	IO	25
13.86	OD	Stellar	IO	90
13.86	OS	Stellar	IO/EO	26/68
13.106	OD	Stellar	IO/EO	20/64
13.106	OS	Stellar	IO/EO	20/64
150,157	OS	Stellar	IO/EO	22/12
150,069	OD	Stellar	IO/EO	67/88
150,110	OD	Stellar	IO/EO	247/88
150,171	OD	Stellar	EO	86
150,171	OS	Stellar	IO/EO	376/86
13.171	OD	Stellar	IO/EO	124/89
13.171	OS	Stellar	EO	89
13.264	OD	Stellar	EO	39
13.264	OS	Stellar	EO	39

Gray and white bars represent different animals.

### IOP telemetry systems

**Figure 4:**
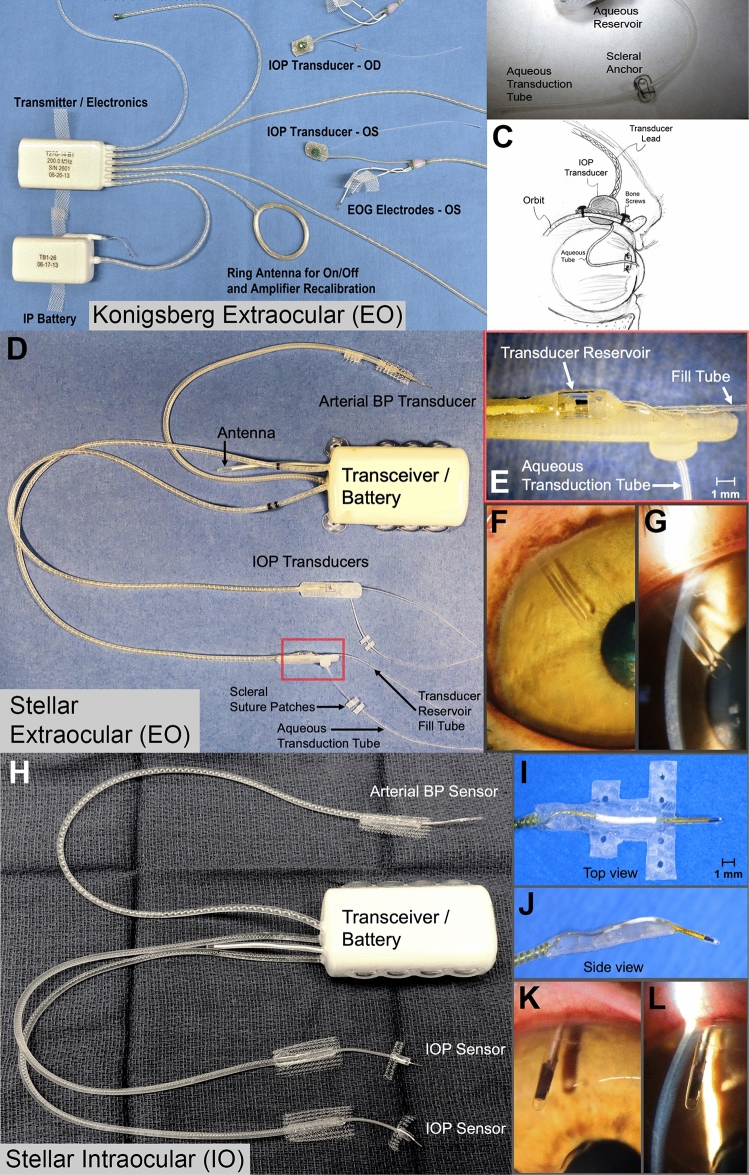
**(Top)** Konigsberg Extraocular (EO) System; (**A)** Photograph of the second-generation Konigsberg Instruments T27G total implant system for continuous monitoring of bilateral IOP (extraocular), bilateral electrooculogram (EOG), aortic blood pressure, and body temperature. **(B and C)** A 23-gauge silicone tube delivers aqueous from the anterior chamber to a fluid reservoir on the intra-orbital side of the transducer; the tube (with appropriate slack to allow for eye movement) is trimmed, inserted into the anterior chamber, sutured to the sclera using the integral scleral anchor plate, and covered with a corneal patch graft (not shown). (^6^^27^Adapted from Downs* et a*l. with permission.) **(Middle)** TSE-Stellar Extraocular (EO) System; (**D**) Photograph of the TSE-Systems Stellar Bilateral IOP/BP extraocular total implant system; (**E**) A 23-gauge silicone tube delivers aqueous from the anterior chamber to an extra-orbital fluid reservoir containing a piezoelectric IOP transducer; the tube (with appropriate slack to allow for eye movement) is trimmed, inserted into the anterior chamber and sutured to the sclera using integral suture patches (**F**) *En face* photograph of the tube in the anterior chamber; (**G**) Slit lamp photograph of the intraocular placement of the tube in the anterior chamber relative to the cornea and iris. **(Bottom)** TSE-Stellar Intraocular (IO) System; (**H**) Photograph of the TSE-Systems Stellar Bilateral IOP/BP intraocular total implant system; (**I)** Top view of the IOP transducer and integrated scleral baseplate for affixing the transducer to the eye wall under Tenon’s capsule and conjunctiva; (**J**) Side view of the IOP transducer and integrated scleral baseplate; (**K**) *En face* photograph of the piezoelectric IOP transducer in the anterior chamber; (**L**) Slit lamp photograph of the intraocular placement of the piezoelectric IOP transducer in the anterior chamber relative to the cornea and iris. (Adapted from Jasien et al.^27^ with permission).

The Konigsberg EO system measured IOP continuously at 500 Hz, whereas the Stellar EO and IO systems measured IOP at 200 Hz at a 10% duty cycle (15 s out of every 150 s); all Stellar data were interpolated to estimate continuous sampling for direct comparison to the previously published Konigsberg data. Stellar data interpolation for the frequency of transient IOP fluctuations in each hour was accomplished by dividing the frequency by the percentage of time in each hour for which data were available after filtering for noise and dropout. Interpolation was not needed for the portion of total IOP due to transient IOP fluctuations, which is a relative percentage that does not scale with time. The TSE-Stellar and Konigsberg extraocular systems consist of a tube that is placed directly in the anterior chamber that is attached to a fluid-filled reservoir containing the transducer that is placed in the orbit (Fig. 4). Whereas the TSE-Stellar intraocular system consists of the transducer being placed directly in the anterior chamber (Fig. 4). Both of these systems have been validated and assessed in various research capacities related to IOP and transient IOP fluctuations^6–8,24–28^. All IOP transducers were calibrated bi-weekly via anterior chamber manometry, and data were adjusted for drift between calibrations and barometric pressure in real time. Animals were anesthetized via intramuscular injections of ketamine (3 mg/kg) with dexmedetomidine (50 mcg/kg) followed by isoflurane inhalant anesthesia (1–3%) for maintenance.

### Data collection and analysis: finite impulse response filtering to identify transient IOP fluctuations

**Figure 5:**
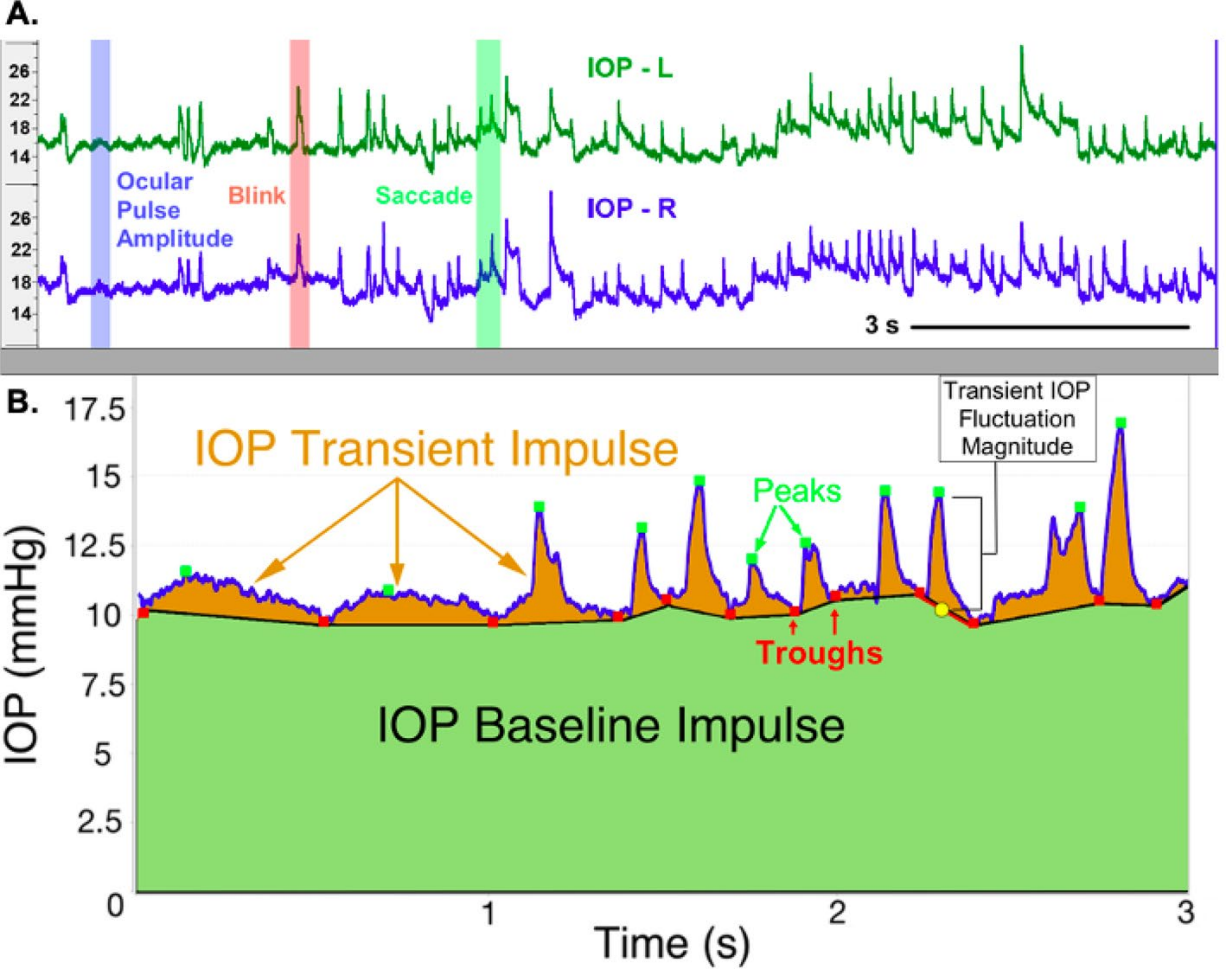
Identification and quantification of frequency, magnitude, and impulse of transient IOP fluctuations. **(A)** The top panel shows a screenshot of ~ 11 s of continuous telemetry data showing IOP signals from the left (L) and right (R) eyes of an animal. Selected verified blinks, saccades, and ocular pulse amplitude events are shown with red, green, and blue shading, respectively. **(B)** The bottom panel shows the area under the IOP vs. time curve, which represents total IOP impulse, a surrogate measure for the cumulative IOP-related mechanical energy the eye must withstand and absorb over time. The green-shaded area represents the IOP Baseline Impulse, which is a measure of the IOP energy the eye must withstand due to momentary baseline IOP and the orange-shaded area represents the IOP Transient Impulse, which is a measure of the IOP energy the eye must withstand due to transient IOP fluctuations alone. Used and^8,28^ adapted from Turner et al and Markert et al. with permission.

The frequency and magnitudes of transient IOP fluctuations above momentary baseline were quantified using an automated finite impulse response (FIR) filtering system. As previously used by our group, we quantified transient IOP by running the raw IOP data through a 7-sample, 14 ms running average to remove noise (Fig. 5)^8,28^. As previously described by Markert et al., a dual-band finite FIR filter in both a low- and high-frequency range was then used to identify transient IOP fluctuation peaks and troughs. The low-band FIR filter (0.5–3 Hz) identifies the peaks and troughs of transient IOP fluctuations 0.3–2 s in duration, whereas the high-band FIR filter (5–10 Hz) identifies high-frequency transient IOP fluctuations of 0.1–0.3 s duration. Transient IOP fluctuation magnitude was quantified as the difference between the largest IOP value (peak) relative to the adjacent two troughs that were averaged into a single baseline IOP value. The frequency of transient IOP fluctuation magnitudes were analyzed by magnitude bin, the smallest of which was determined by our minimum transient magnitude detection resolution (0.6 mm Hg) and analyzing the ocular pulse amplitude (typically less than 2 mm Hg). The other magnitude bins were defined arbitrarily to represent small (2 to 5 mm Hg), medium (5 to 10 mm Hg), large (10 to 15 mm Hg), and very large (> 15 mm Hg) transient IOP fluctuations^8^. Following the counting and sorting of raw data by magnitude, the frequency of transient IOP fluctuations in each magnitude bin were averaged hourly for each day by eye, then plotted and analyzed across all days. The IOP transducers of all systems have a measurement noise of ± 0.2 mmHg, therefore we set the lower limit of transient IOP fluctuation magnitudes reported at 0.6 mmHg. The total IOP impulse, defined as the area under the continuous IOP versus time curve, is an engineering-based metric that serves as a surrogate measure of the total amount of IOP-related mechanical energy the eye must withstand over time. IOP transient impulse is quantified as the portion of total IOP impulse associated with transient IOP fluctuations alone. IOP transient impulse is a surrogate measure of the energy the ocular coat must absorb due to only transient IOP fluctuations. The relative contribution of IOP transient impulse, calculated as a percentage of total IOP impulse, was used to assess the amount of transient IOP fluctuation-related energy the eye must absorb over time relative to the total IOP-related energy^8^.

### Statistical analyses

We used linear mixed effects models to assess the differences in the mean number of transient IOP fluctuations per hour greater than 0.6 mmHg and > 15 mmHg in magnitude, as well as the mean relative contribution of transient IOP fluctuations to total IOP, between system types (Konigsberg EO, Stellar EO and Stellar IO), NHPs, eyes and the sleeping versus waking periods. An identical analysis was performed in the subset of eyes in which both the Stellar IO and EO systems were implanted in series, in order to confirm that results from the larger analysis was valid.

## Data Availability

The datasets generated and analyzed in the current study are available from the corresponding author by reasonable request.
